# Nursing Affairs in Ireland

**Published:** 1917-12-22

**Authors:** 


### NURSING AFFAIRS IN IRELAND.

### Miss Brodrick takes the Field.

The Hon. Albinia Brodrick was the principal speaker at the first meeting of the Irish Nursing Board, held in Dublin on Friday of last week. Miss Brodrick is an interesting personality. She is a sister of Lord Midleton, clever and cultured, a trained nurse running a small and useful hospital, with a little farm attached, in rural Kerry, some thirty miles from a railway station, and affects a Puritan eccentricity of rustic dress. She has some reputation as a speaker, which may be justified. The best lecturers vary in their effectiveness, doubtless according to their theme, and it might be scarcely fair to judge her ability in this respect from Friday's distinctly dull and platitudinarian performance.

Miss Brodrick appeared as the champion of the newly formed Irish Nursing Board, which, it will be remembered, set out to found in Ireland an institution for nurses modelled on the pattern of the College of Nursing. This description is taken from the printed circular signed by the hon. secretary, Miss Carson Rae. The lecturer's mission, therefore, was to demonstrate to the profession why they should hold to the imitation and despise the model, both being Irish and in their midst. The task was difficult, and Miss Brodrick faced it with the air of one who realised the weakness of her case.

The outstanding features of the College of Nursing, and consequently of the Irish Nursing Board, are threefold—a Board, an examination scheme, and a Register—and the essential difference between the two institutions is that the College provides for a union of Irish nurses with those of Great Britain, whereas the Irish Nursing Board advocates a policy of isolation. It was naturally to be expected that the lecturer would have concentrated on this issue, being the only issue in which the rank and file of the profession are interested. Instead, she confined her attention to old weather-beaten side-issues, and kept absolutely clear of the broad interests of the nurse. Nor was there any real argument offered concerning even the side-issues, but rather that type of unconvincing pleading which consists of sarcasm, misrepresentation, and what was intended as humour. Here, for example—"there is a thing called the College of Nursing—or, as I might call it, the College of V.A.D.s." This corresponds with a German description of a British victory, and appealed only to Miss Brodrick's fellow-propagandists, and not at all to the puzzled nurse who is unable to distinguish between the coin and the counterfeit. Miss Brodrick dwelt much on the "government" of nurses, and expressed her intense objection to nurses being "governed" from England, "legislated for" from England, and so forth, all of which was, of course, platform trickery and an appeal to political rather than to nursing affairs. I am not aware that nurses want to be "governed" at all, either in England or in Ireland, but the Irish Nurses' Association, out of which the Irish Nursing Board has grown, and of which Miss Brodrick was a distinguished member, never did anything else than "govern" the Irish nurse. Neither she nor they can point to one single forward movement in the nursing world which they have either achieved or initiated.

A curious inconsistency of the lecturer, which may be explicable, but which I am unable to understand, is that, having delivered a tirade against English "impertinence" in founding an institution for nurses in this country, Miss Brodrick made frequent reference to lectures which she had recently delivered in England and in Scotland, and likewise to the envy which English and Scottish nurses feel because Ireland possesses what they so badly need, a real live Nursing Board. One wonders to what extent on those occasions she expressed her sense of abhorrence for everything that had its origin outside Ireland.

So much for the lecture. At its conclusion the chairman announced that the meeting was open for discussion. The chairman was an ideal president. He is the identical gentleman who presided when Miss Cox-Davies and Miss Rundle addressed the Dublin nurses, and who then took advantage of his position to heckle these ladies because they came from England. Many persons present were looking forward to an opportunity of replying to Miss Brodrick, but by a pre-concerted stratagem half a dozen members of the Irish Nursing Board, with the utmost bad taste, trooped one by one to the platform and talked the meeting out. Not one of them uttered an interesting or original remark. They simply talked until the pressmen present one by one slipped away, when Dr. O'Carroll observed that as the press representatives had vanished there was nothing to be gained by prolonging the proceedings.

I can conceive nothing more truly pathetic than the nursing situation in Ireland. It is notorious that English and Scottish nurses are immeasurably in advance of Irish nurses in pay, working hours, privileges, and conditions of service generally; and now these women, preaching democracy and practising autocracy, have succeeded in adding to the difficulties that lie in the way of reform by creating faction. We have now in consequence three camps, but concerning the Irish League of Trained Nurses I have every hope, although at the moment they are busily engaged in pursuing a myth. They are absolutely and completely democratic. They are in revolt against the autocracy of the Irish Nursing Board, and petitioned them to abolish their six-year unelected tenure of office. They are most of them simple nurses seeking the light, and I believe that they will find it sooner or later. Their attitude towards the College is one of suspicion. They fear that in some way they are going to be "done" if they join.

There are one or two facts that I should like to put before the Irish nurses of the rank and file. I am not in the least degree afraid of the word trade-union. The essential difference between an ordinary trade-union and a nurses' union is the strike. Nurses could never, and would never, in the worst possible circumstances, in any country desert their patients. Leaving this aside as unthinkable and impossible, a union of nurses is a trade-union. I wish further to point out that every Irish trade union worth considering is affiliated with English and Scottish trade unions, and it is scarcely necessary to say that this attitude is not adopted for sentimental reasons. The nursing profession of the United Kingdom will never and can never accomplish anything for themselves unless they are united. There are said to be some seventy thousand nurses of the rank and file. What could they not accomplish if they formed one solid phalanx, instead of being led hither and thither by self-seeking propagandists of faction! Speaking for Ireland I am quite satisfied that the rank and file of the profession are being fooled. Their professed leaders have four war cries—Patriotism, Lay Interference, the V.A.D.s, and English Control.

